# The Traditional Chinese Medicine Formula FTZ Protects against Cardiac Fibrosis by Suppressing the TGF*β*1-Smad2/3 Pathway

**DOI:** 10.1155/2022/5642307

**Published:** 2022-04-19

**Authors:** Yue Zhang, Dongwei Wang, Kaili Wu, Xiaoqi Shao, Hongtao Diao, Zhiying Wang, Mengxian Sun, Xueying Huang, Yun Li, Xinyuan Tang, Meiling Yan, Jiao Guo

**Affiliations:** ^1^Center for Drug Research and Development, Guangdong Pharmaceutical University, Guangzhou 510006, China; ^2^Guangdong Metabolic Diseases Research Center of Integrated Chinese and Western Medicine, Guangzhou 510006, China; ^3^Key Laboratory of Glucolipid Metabolic Disorder, Ministry of Education of China, Beijing, China; ^4^Institute of Chinese Medicine, Guangdong Pharmaceutical University, Guangzhou 510006, China; ^5^Guangdong TCM Key Laboratory for Metabolic Diseases, Guangzhou 510006, China

## Abstract

**Background:**

Fu fang Zhen Zhu Tiao Zhi (FTZ) is a patented preparation of Chinese herbal medicine that has been used as a natural medicine to treat several chronic diseases including cardiovascular disease. However, its effects on cardiac fibrosis remain unclear. Therefore, this study was designed to investigate the effects and potential mechanisms of FTZ in treating cardiac fibrosis.

**Methods:**

FTZ was administered to mice by oral gavage daily at a dosage of 1.2 g/kg or 2.4 g/kg of body weight for 7 weeks after a transverse aorta constriction (TAC) surgery. Doppler echocardiography, hematoxylin and eosin staining, and Masson's trichrome staining were used to assess the effect of FTZ on the cardiac structure and function of mice that had undergone TAC. EdU and wound-healing assays were performed to measure the proliferative and migratory abilities of cardiac fibroblasts. Western blotting and qRT-PCR were used to determine the expression of TGF*β*1, Col1A2, Col3, and *α*-SMA proteins and mRNA levels.

**Results:**

FTZ treatment reduced collagen synthesis, attenuated cardiac fibrosis, and improved cardiac function in mice subjected to TAC. Moreover, FTZ treatment prevented the proliferation and migration of cardiac fibroblasts and reduced Ang-II-induced collagen synthesis. Furthermore, FTZ downregulated the expression of TGF*β*1, p-smad2, and p-smad3 and inhibited the TGF*β*1-Smad2/3 pathway in the setting of cardiac fibrosis.

**Conclusion:**

FTZ alleviated the proliferation and migration of cardiac fibroblasts and suppressed collagen synthesis via the TGF*β*1-Smad2/3 pathway during the progression of cardiac fibrosis. These findings indicated the therapeutic potential of FTZ in treating cardiac fibrosis.

## 1. Introduction

Cardiovascular disease continues to be the leading cause of death worldwide [1]. Heart failure (HF) is the common clinical manifestation of the advanced stages of many cardiac diseases. Several factors including cardiac fibrosis contribute to HF. As an intricate progression, cardiac fibrosis is characterized by adverse cardiac structural remodeling, which may eventually lead to HF [2, 3].

Fu fang Zhen Zhu Tiao Zhi (FTZ) is an effective traditional Chinese herbal preparation predominantly composed of eight Chinese herbs with definite curative effects and without obvious toxic or side effects. It has been used for over 10 years in a clinical setting to treat nonalcoholic fatty liver disease [4], atherosclerosis, diabetes [5, 6], aging [7, 8], and disorders of glucose and lipid metabolism. Results from our preliminary *in vivo* studies demonstrated that FTZ could ameliorate several pathological processes such as inflammation, abnormal blood coagulation, endothelial dysfunction, and the formation of atherosclerotic plaques. In addition, FTZ can regulate glucose and lipid metabolism and reduce oxidative stress in rodent models [9–11]. More importantly, a recent study reported that FTZ could ameliorate diabetic cardiomyopathy by inhibiting inflammation and cardiac fibrosis [12]; however, its mechanism in inhibiting cardiac fibrosis was unclear. Given the universality of FTZ, some of the individual herbs in FTZ including glossy privet fruit [13], *Atractylodes* [14], *Coptis* [15, 16], and pseudoginseng [17, 18] have been traditionally used to treat fibrosis. Thus, in this study, we examined the effects of FTZ on the heart and explored the therapeutic effect of FTZ in cardiac fibrosis.

Several studies suggest that chronic hypertension might lead to cardiac pressure overload, which contributes to the progression of cardiac fibrosis. Besides, it has been reported that hormones and growth factors, such as angiotensin II (Ang-II) and transforming growth factor-*β*1, could promote the activation of cardiac fibroblasts (CFs), causing an increase in *α*-SMA positive cells. Excess activation of CFs might eventually cause cardiac fibrosis and dysfunction due to the secretion of abundant extracellular matrix (ECM) [19, 20]. During this process, Ang-II elevates TGF*β*1 expression, which subsequently mediates the phosphorylation of Smad2 and Smad3. Activated TGF*β*1-Smad2/3 signaling upregulates the levels of various target genes including Col1A2 and Col3. These findings suggest that suppressing the TGF*β*1-Smad2/3 pathway might help inhibit the activated CFs and alleviate cardiac fibrosis [21, 22].

In this study, we demonstrated that FTZ could not only improve cardiac dysfunction but also ameliorate cardiac fibrosis. Additionally, we performed a series of experiments using cardiac-fibrotic models and found that FTZ was effective in treating cardiac fibrosis, and its molecular mechanism involved regulation of the TGF*β*1-Smad2/3 pathway.

## 2. Methods and Materials

### 2.1. Preparation of FTZ

Eight kinds of Chinese medicinal herbs in FTZ (*Citri sarcodactylis fructus*, *Ligustri lucidi fructus*, *Salviae miltiorrhizae radix et rhizoma*, *Notoginseng radix et rhizoma*, *Coptidis rhizoma*, *Atractylodis macrocephalae rhizoma*, *Cirsii japonici herba et radix*, and *Eucommiae cortex*) were purchased from Zhixin Chinese Herbal Medicine Co. Ltd. (Guangzhou, China) and identified by Professor Wei He and Senior Lecturer Li Yong, Guangdong Pharmaceutical University. The preparation of FTZ was consistent with the protocol described previously [23]. And the quality control of FTZ was performed by UPLC-MS/MS as previously reported [24]. The FTZ used in this study was from the First Affiliated Hospital of Guangdong Pharmaceutical University.

### 2.2. Animals and Treatment

Male C57BL/6 mice (6–8 weeks old) weighing 20–22 g were purchased from Changzhou Cavens Laboratory Animal Co. Ltd., China. Mice were housed in cages, provided a chow diet, and subjected to a 12 h light/12 h dark cycle in a room maintained at standard conditions (temperature 25 ± 1°C; humidity 55 ± 5%). All experiments were approved by the Animal Research Ethics Committee of Guangdong Pharmaceutical University. Protocols used for transverse aorta constriction (TAC) and sham operation were from previously reported studies [25]. Briefly, a TAC operation was performed for the partial ligation of the transverse aorta using a 6-0 suture that was banded over a 27-gauge needle. Mice were randomly assigned to 5 groups. A sham operation was performed on one of the groups of mice and TAC on the others. On day 7 after the operation, among the TAC mice, a quarter of the mice were administered captopril (intragastrically, 10 mg/kg/day), and the others were treated with either a low or a high dose (intragastrically, 1.2 g/kg/day or 2.4 g/kg/day) of FTZ or the vehicle for 7 weeks. FTZ dose was selected based on that used for humans clinically. Captopril dosage was determined based on that used in previous studies [2].

### 2.3. Echocardiography

After 7 weeks of drug administration, echocardiography was conducted using a Vevo 2100 system (VisualSonics, Canada) with a high-frequency (30 MHz) MS-400 transducer. Mice were anesthetized under isoflurane inhalation (1%). Cardiac indices were measured and calculated using computer algorithms. All measured cardiac indices are presented as the mean of 3 consecutive cardiac cycles.

### 2.4. Histopathology and Immunohistochemistry

Cardiac tissues were fixed in phosphate-buffered saline with 4% paraformaldehyde for 24 h at room temperature. Next, tissues were embedded in paraffin, and 4 *μ*m-thick sections were prepared for histopathology experiments. Hematoxylin and eosin (H&E), Masson's trichrome, and Sirius red staining were used following the manufacturers' instructions to observe the changes in cardiac morphology and determine the extent of fibrosis. In addition, cardiac tissues were stained with *α*-smooth muscle actin (*α*-SMA; Proteintech, 1:2,000) or TGF*β*1 (Proteintech, 1:500) antibodies at the same region of every heart slice. The *α*-SMA and TGF*β*1 positive area and the fibrotic area were quantified by calculating the percentage of collagen staining using ImageJ analysis.

### 2.5. Cell Culture

CFs were isolated from 1–3 day old neonatal mice, cultured in Dulbecco's modified Eagle medium (supplemented with 10% fetal bovine serum and 1% penicillin/streptomycin) at 37°C in an atmosphere of 5% CO_2_ and 95% air. CFs were stimulated with FTZ (50 *μ*g/mL or 100 *μ*g/mL) and Ang-II (100 nM). The concentrations of FTZ and Ang-II were determined by referring to previous studies [11, 26, 27].

### 2.6. Wound-Healing Assay

Cells were cultured overnight in six-well plates until they reached 95% confluence. Wound-healing assay was conducted by creating a scratch wound with a 0.1–20 *μ*L pipette tip. Next, CFs were stimulated with Ang-II (100 nM) or/and FTZ (50 *μ*g/mL or 100 *μ*g/mL). Changes in scratches were observed at 24 and 48 h, and photographs were captured.

### 2.7. Cell-Proliferation Assay

After 24 h of treatment with FTZ and Ang-II, 10 *μ*L of cell counting kit-8 (CCK-8) reagent was added to each well and incubated in the culture medium at 37°C for 3 h. The optical density of each well was obtained at 450 nm.

### 2.8. EdU-Proliferation Assay

After drug treatment, CFs were incubated with 10 *μ*M EdU at 37°C for 3 h. Next, cells were fixed in 4% paraformaldehyde and treated with 0.3% Triton X-100 for 15 min, respectively, and stained according to the manufacturers' instructions. Cell proliferation was observed, and images were photographed using a fluorescence microscope (Olympus Optics, Tokyo, Japan). The cell proliferation was calculated by using ImageJ software.

### 2.9. Western Blotting

Total proteins were isolated from CFs using RIPA lysis buffer supplemented with a protease/phosphatase inhibitor. Proteins were fractionated using sodium dodecyl sulfate-polyacrylamide gel electrophoresis and transferred to nitrocellulose (NC) membranes. After blocking with 5% nonfat milk or BSA, the NC membranes were incubated with the corresponding antibodies of the target proteins. The antibodies included p-smad2 (AbSci, 1:500), smad2 (Proteintech, 1:1,000), p-smad3 (AbSci, 1:500), smad3 (Proteintech, 1:1,000), TGF*β*1 (Proteintech, 1:1,000), Col1A2 (Proteintech, 1:2,000), Col3 (Proteintech, 1:500), *α*-SMA (Proteintech, 1:20,000), and *β*-actin (Proteintech, 1:2,000). After overnight incubation at 4°C, the NC membranes were incubated with the secondary antibody (1:8,000) for 50 min at room temperature. The protein bands were scanned and analyzed using the Odyssey Imaging System.

### 2.10. Reverse Transcription and Quantitative Real-Time Polymerase Chain Reaction (RT-qPCR)

Total RNA was extracted from CFs or cardiac tissues using TRIzol following the manufacturer's protocol and then reverse-transcribed to obtain cDNA. qRT-PCR was performed to detect mRNA levels of target genes using SYBR Green Real-Time PCR Master Mix. The 2−^∆∆CT^ method was used to present the changing levels of target genes, which were normalized to *β*-actin mRNA.

### 2.11. Statistical Analysis

All values are expressed as the mean ± SEM. Student's *t*-test was used for comparison between groups and one-way ANOVA for comparison of multiple groups. *P* < 0.05 was considered to indicate a significant difference. GraphPad Prism 7.0 was used to analyze all statistical data.

## 3. Results

### 3.1. FTZ Prevents Pressure Overload-Mediated Cardiac Dysfunction in Mice

**Figure 1:**
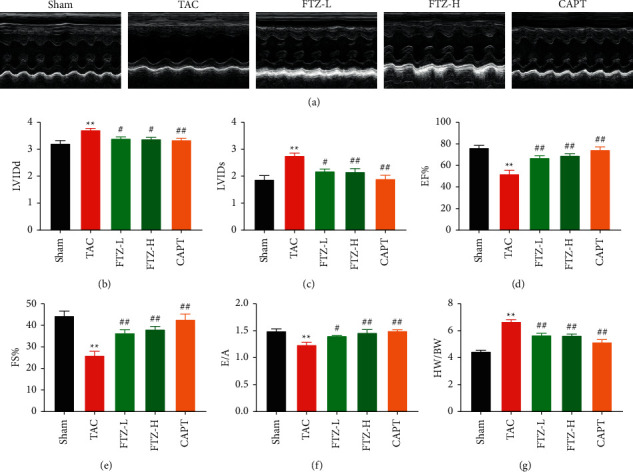
FTZ improves cardiac dysfunction and alleviates cardiac hypertrophy in pressure overload-mediated cardiac dysfunction in mice (a) Representative M-mode echocardiography of the left ventricular chamber. FTZ-L and FTZ-H represent doses of 1.2 and 2.4 g/kg FTZ, respectively. CAPT represents the 10 mg/kg captopril group. (b–f) Echocardiographic assessment of LVIDd, LVIDs, EF%, FS%, and E/A; *n* = 8 per group. (g) HW/BW ratios in each group; *n* = 8 per group. Data are presented as the mean ± SEM. ^*∗∗*^*P* < 0.01 versus the sham group and ^*#*^*P* < 0.05 and ^*##*^*P* < 0.01 versus the TAC group.

To determine the influence of FTZ on cardiac function in mice, we performed a TAC operation and assessed the cardiac diastolic and systolic function using echocardiography. Oral administration of FTZ or the positive control, captopril, for 7 weeks sufficiently improved cardiac function, as evidenced by decreased left ventricular internal diameter at end-diastole (LVIDd) and left ventricular internal diameter at end-diastole (LVIDs; Figures 1(a)–1(c)). The increased ejection fraction (EF) and fractional shortening (FS) of mice that underwent TAC and were administered FTZ suggested that FTZ could ameliorate cardiac systolic function (Figures 1(d)–1(e)). Moreover, TAC-induced diastolic dysfunction, as indicated by a decrease in the E/A peak ratio, was also reversed in FTZ-treated mice (Figure 1(f)). Moreover, decreased heart weight/body weight (HW/BW) indicated that FTZ could attenuate TAC-induced myocardial hypertrophy in response to pressure overload (Figures 1(g) and S1). Therefore, these data indicated that FTZ could ameliorate TAC-induced cardiac systolic and diastolic dysfunction.

### 3.2. FTZ Attenuates Cardiac Fibrosis in Mice

**Figure 2:**
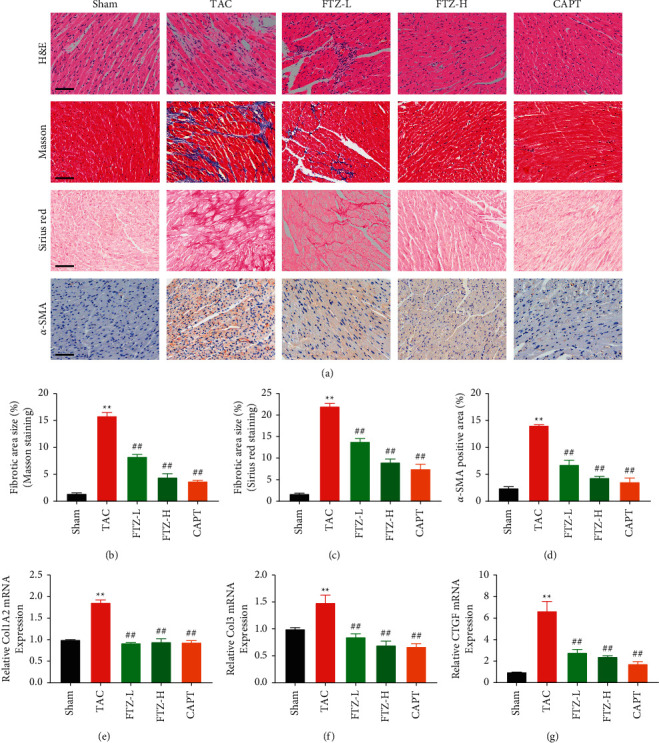
FTZ reduces cardiac fibrosis in pressure-overload mice. (a–d) H&E staining results of cardiac tissue are shown. Scale bars: 50 *μ*m. *n* = 3 per group. Masson's trichrome staining results of cardiac tissue are shown. Scale bars: 50 *μ*m. *n* = 3 per group. Fibrosis of cardiac tissues stained with Sirius Red. Scale bars: 50 *μ*m. *n* = 3 per group. Immunohistochemical detection of *α*-SMA. Scale bars, 50 *μ*m. *n* = 3 per group. (e–g) RT-qPCR to determine Col1A2, Col3, and CTGF expression. *n* = 6 per group. Data are presented as the mean ± SEM. ^*∗∗*^*P* < 0.01 versus the sham group and ^*##*^*P* < 0.01 versus the TAC group.

Collagen deposition and cardiac structural changes are the main pathological features of cardiac fibrosis. As seen in Figure 2(a), H&E staining indicated that the structure of myocardial tissues of mice that underwent TAC was destroyed, but the extent of damage to myocardial tissue was reduced in FTZ-treated mice. Collagen deposition in the myocardial tissue of mice subjected to TAC was more obvious than that in the tissues of mice subjected to sham treatment. However, FTZ treatment led to an obvious decline in ECM and collagen synthesis. Immunohistochemical staining showed that *α*-SMA was significantly elevated and distributed in the cardiac tissues of mice subjected to TAC, whereas FTZ treatment significantly reduced *α*-SMA expression. In addition, FTZ also reduced the content of HYP in TAC mice as evaluated by the ELISA kits (Figure S2). The results were consistent with qRT-PCR findings, FTZ significantly downregulated Col1A2, Col3, and connective tissue growth factor (CTGF) mRNA levels compared with those in mice subjected to TAC (Figures 2(e)–2(g)). Collectively, these findings indicated that FTZ might suppress the progression of cardiac fibrosis.

### 3.3. FTZ Inhibits CF Proliferation and Migration *In Vitro*

**Figure 3:**
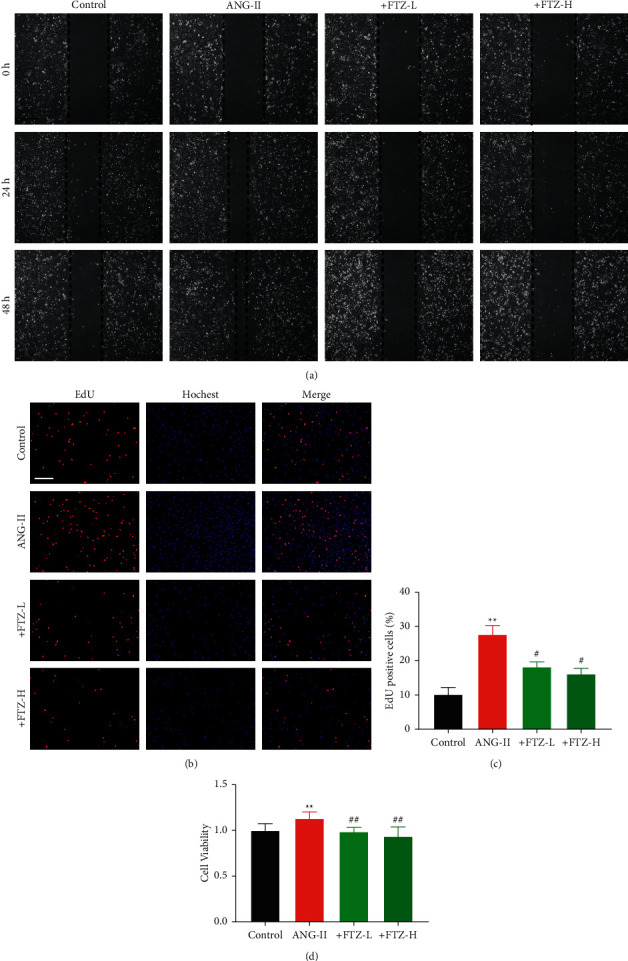
FTZ inhibits the proliferation and migration of cardiac fibroblasts *in vitro*. (a) Wound healing assay shows that FTZ intervention resulted in slow closing of scratch wounds after Ang-II treatment. Scale bars, 200 *μ*m. *n* = 5 per group. (b) and (c) Representative images of EdU staining showing proliferating cells (stained red). Nuclei that are double-labeled with EdU (red) and Hoechst 33342 (blue) were considered to be new proliferative cells. The scale bar indicates 100 *μ*m. *n* = 5 per group. (d) Effects of FTZ on cell viability were measured using a CCK-8 assay. *n* = 8 per group. Data are presented as the mean ± SEM. ^*∗∗*^*P* < 0.01 versus the control group and ^#^*P* < 0.05 and ^*##*^*P* < 0.01 versus the Ang-II group.

Some studies have shown that inhibiting proliferation, migration, and collagen deposition in CFs can prevent or even reverse cardiac fibrosis [20, 28]. To determine whether FTZ had a direct effect on CFs with Ang-II, we conducted a wound-healing assay and found that FTZ could inhibit Ang-II-induced CF migration (Figure 3(a)). EdU and CCK-8 assays showed that FTZ could significantly reduce the proliferative ability of CFs. Compared with the CFs treated with Ang-II, those treated with FTZ showed a marked decrease in proliferation with any effect on cell viability (Figures 3(b)–3(d) and S3). In addition, the weakened apoptosis program and abnormal apoptosis mechanism of cardiac fibroblasts are the main reasons for the further development of cardiac fibrosis [29]. As shown in Figure S4, FTZ could restore the normal apoptosis program. These results suggested that FTZ could inhibit abnormal proliferation and migration in CFs.

### 3.4. FTZ Reduces Collagen Synthesis *In Vitro*

**Figure 4:**
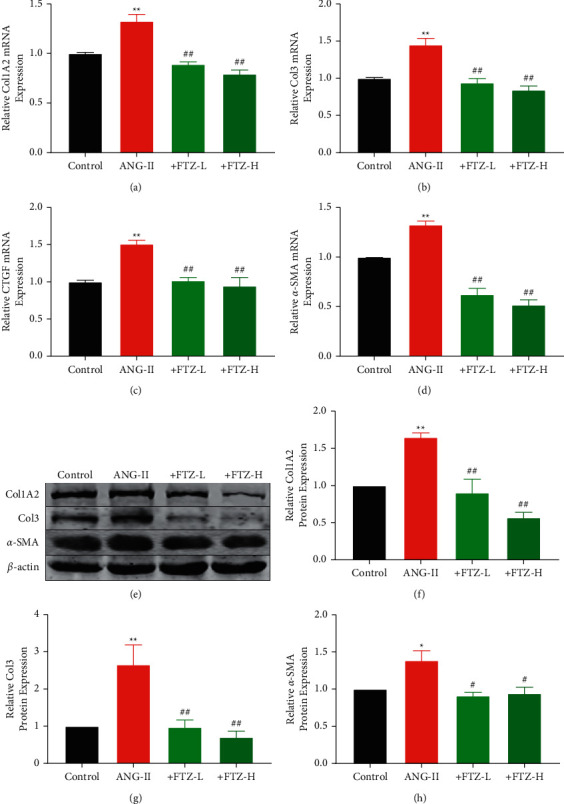
FTZ reduces collagen synthesis *in vitro.* (a)–(d) RT-qPCR to determine the expression of Col1A2, Col3, CTGF, and *α*-SMA. *n* = 5 per group. (e)–(h) Col1A2, Col3, and *α*-SMA expression were determined using western blotting. *n* = 5 per group. Data are presented as the mean ± SEM. ^*∗*^*P* < 0.05 and ^*∗∗*^*P* < 0.01 versus the control group and ^*#*^*P* < 0.05 and ^*##*^*P* < 0.01 versus the Ang-II group.

Under pathological conditions, the abnormal migration and proliferation of CFs could promote excessive collagen secretion. Furthermore, abnormal accumulation of collagen can reduce myocardial compliance and increase myocardial hardness, which eventually leads to cardiac dysfunction during systole and diastole [19, 30]. This outcome is characterized by elevated levels of Col1A2, Col3, *α*-SMA, and CTGF. Therefore, changes in the expression of these fibrosis-related genes were determined. We found that FTZ could significantly inhibit the mRNA levels of Col1A2, Col3, *α*-SMA, and CTGF after induction with Ang-II (Figures 4(a)–4(d)). Consistently, the protein expression of Col1A2, Col3, and *α*-SMA was significantly decreased in CFs treated with FTZ compared with those treated with Ang-II (Figures 4(e)–4(h)). Furthermore, we evaluated the protein expression level of matrix metalloproteinases (MMPs), and we found that FTZ could reduce the expression of MMP1 and MMP2 (Figure S5). Overall, our findings suggested that FTZ could reduce collagen synthesis in CFs.

### 3.5. FTZ Attenuates Cardiac Fibrosis by Downregulating the TGF*β*1-Smad2/3 Pathway

**Figure 5:**
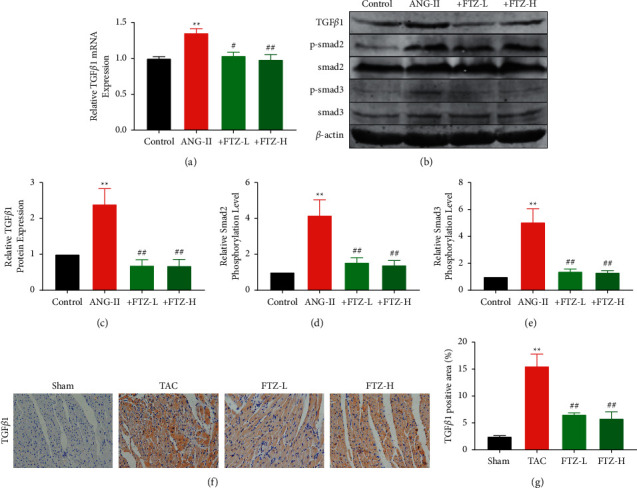
FTZ improves cardiac fibrosis by inhibiting the TGF*β*1-Smad2/3 pathway. (a) mRNA expression of TGF*β*1 was determined using RT-qPCR. *n* = 5 per group. (b–e) Protein expression of TGF*β*1, p-smad2, smad2, p-smad3, and smad3 were determined using western blotting. *n* = 5 per group. Data are presented as the mean ± SEM. ^*∗∗*^*P* < 0.01 versus the control group and ^##^*P* < 0.01 versus the Ang-II group. (f) and (g) TGF*β*1 expression of consecutive sections of cardiac specimens assessed using immunohistochemical staining. Scale bar: 50 *μ*m. *n* = 3 per group. Data are presented as the mean ± SEM. ^*∗∗*^*P* < 0.01 versus the sham group and ^*##*^*P* < 0.01 versus the TAC group.

TGF*β*1-Smad2/3 signaling participates in the progression of cardiac fibrosis and is considered a classical pathway in regulating cardiac fibrosis [21]. In this study, we determined the markers of the TGF*β*1-Smad2/3 signaling pathway to investigate the potential mechanisms of FTZ in preventing cardiac dysfunction and cardiac fibrosis. We found that the expression of TGF*β*1, p-smad2, and p-smad3 increased significantly after Ang-II induction; however, their expression decreased considerably after FTZ treatment (Figures 5(a)–5(e)). Moreover, immunohistochemical staining showed that the TGF*β*1 was obviously upregulated and distributed in the myocardial tissue in mice subjected to TAC (Figure 5(f)). To better clarify the mechanism, we used the TGF*β*1 agonist; we found that TGF*β*1 agonist reversed FTZ inhibition of cardiac fibroblast activation (Figure S6). These results indicated that FTZ was effective in inhibiting the TGF*β*1-Smad2/3 pathway in mice with cardiac fibrosis.

## 4. Discussion

Cardiac fibrosis is a common pathological phenomenon in cardiovascular disease that is characterized by excessive ECM deposition [31]. Cardiac fibrosis destroys the normal structure of the heart muscle, leading to myocardial dysfunction, electrical activity and mechanical impairment, and acceleration of HF progression [32]. Although current treatment strategies can be used to improve the clinical symptoms of patients with HF, it is difficult to reverse the pathological process of cardiac fibrosis, and its severity is closely related to the long-term mortality of patients. Therefore, the diagnosis, prevention, and treatment of cardiac fibrosis are important goals in the management of HF.

In recent years, traditional Chinese medicine has been widely used in many countries to treat various diseases including cardiovascular diseases [33, 34]. FTZ is a representative prescription of the “Tiaogan Qishu Huazhuo” theory, which is summarized based on the clinical practice of more than 10 years [35]. The liver plays a vital role as a regulator by coordinating with multiple organs. The function of the heart is to regulate blood flow and circulation. One of the main functions of the liver is blood storage (Xin Zhu Xing Xue Er Gan Zhu Cang Xue). In addition, the heart regulates spiritual activities and, via regulating the liver, opens the central system, dredges the “qi” of the whole body, and makes it flow smoothly (Xin Cang Shen Er Gan Zhu Shu Xie). Therefore, the relationship between the heart and the liver is mainly manifested in two aspects: blood circulation and storage and mental regulation. Abnormal liver function can affect the storage of blood, which in turn can affect cardiac function and lead to arrhythmias. The prophylactic use of FTZ could alleviate stress and pressure overload on the heart by regulating the liver as well as by improving the poor flow of “qi” and blood resulting from the blockage of blood flow, thereby improving cardiac function. Studies have reported that FTZ mainly affects lipid metabolism, glucose metabolism, and other metabolic pathways, and has significant efficacy in diabetes, atherosclerosis, nonalcoholic fatty liver disease, and the improvement of insulin resistance and other disorders of glucose and lipid metabolism [6, 23]. Among the components of FTZ, glossy privet fruit, *Atractylodes*, *Coptis*, and pseudoginseng have been reported to have therapeutic effects on fibrosis. Therefore, we speculated that FTZ might play an important role in the treatment of cardiac fibrosis. Our results showed that FTZ could significantly improve cardiac function and inhibit collagen deposition in mice subjected to TAC and also inhibit Ang-II-induced proliferation and migration of fibroblasts *in vitro*.

Cardiac fibrosis results from the continuous and repeated aggravation of myocardial ischemia and hypoxia caused by severe atherosclerotic stenosis of the coronary artery. Currently, there is no effective approach to curing cardiac fibrosis. Captopril is an angiotensin-converting enzyme inhibitor commonly used to treat hypertension, HF, and cardiac fibrosis [36, 37]; however, we found that FTZ might have more benefits than captopril. Patients with cardiac fibrosis often suffer from atherosclerosis, diabetes, and abnormal lipid metabolism, and FTZ has a therapeutic effect on these diseases. Therefore, FTZ shows potential for the treatment of cardiac fibrosis.

Under normal conditions, the synthesis and degradation of extracellular matrix in the myocardium are in a dynamic balance. Among them, matrix metalloproteinases are the important material basis to maintain this equilibrium state, which are responsible for the ECM degradation [38]. In a pathological state, the dynamic balance of ECM is broken that results in ECM depositions. After ANG-II treatment, the collagens contents in cardiac fibroblasts were increased. In order to maintain a balance between the production and degradation of the extracellular matrix, the levels MMPs were also upregulated [39]. Our results showed that after ANG-II treatment, the MMPs expression in cardiac fibroblasts were increased to keep the balance of the extracellular matrix, whereas the symptoms of cardiac fibrosis were relieved by FTZ, and also, the protein expression levels of collagens and MMPs were significantly decreased.

**Figure 6:**
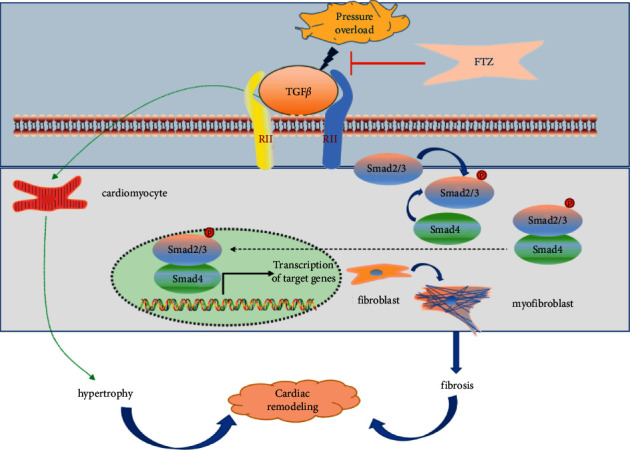
Schematic diagram illustrating the mechanism of FTZ in regulating cardiac fibrosis.

TGF*β*1 plays an important role in the progression of cardiac fibrosis; it can stimulate the proliferation of fibroblasts and induce the expression of growth factors involved in regulating cell proliferation, adhesion, and migration [40]. With higher levels of inflammatory cytokines, the degree of fibrosis is gradually aggravated. Moreover, the damage repair space is smaller, and thus, the process of cardiac fibrosis is irreversible. The Smad family plays a key role in the transduction of TGF*β*1 signals from the cell surface receptors to the nucleus. TGF*β*1 in CFs binds to receptors and activates smad2 and smad3, leading to ECM deposition and cardiac fibrosis [21, 41]. We found that the expression of TGF*β*1, p-smad2, and p-smad3 was significantly increased in the Ang-II group than in the control group; however, the expression in the FTZ-treated group was significantly decreased compared with that in the model group, suggesting that FTZ may play a role in preventing fibrosis by affecting the TGF*β*1-Smad2/3 signal transduction pathway (Figure 6).

Our study has some limitations, and further evaluations are required. For instance, we only investigated the regulatory effect of FTZ on the TGF*β*1-Smad pathway. The detailed molecular mechanism regulated by FTZ was not completely elucidated in this study. Therefore, future investigations are needed to determine how FTZ induces the TGF*β*1-Smad pathway.

## 5. Conclusions

Our results show that FTZ could significantly improve TAC-induced systolic and diastolic dysfunction and reduce cardiac fibrosis in mice, indicating that it could effectively limit myocardial remodeling. Our findings established a novel connection between FTZ and cardiac fibrosis, which enhanced our understanding of the potential of the derivatives of traditional Chinese medicine monomers as effective therapeutics in managing cardiac fibrosis.

## Data Availability

The data used and analyzed during our study are available from the corresponding author on reasonable request.

## Abbreviations

FTZ: Fu Fang Zhen Zhu Tiao Zhi
HF: Heart failure
Ang-II: Angiotensin II
TAC: Transverse aorta constriction
TGF*β*1: Transforming growth factor-*β*1
CFs: Cardiac fibroblasts
EF: Ejection fraction
FS: Fractional shortening
E/A: Transmitral early (E) to atrial (A)
LVIDd: Left ventricular internal diameter at end-diastole
LVIDs: Left ventricular internal diameter at end-systole
HW/BW: Heart weight/body weight
*α*-SMA: *α*-smooth muscle actin
qRT-PCR: quantitative real-time PCR
ECM: Extracellular matrix
OD: Optical density
H&E: Hematoxylin and eosin
CCK: Cell counting kit
NC: Nitrocellulose.

## Supplementary Materials

Supplementary Materials Supplementary Figure 1: FTZ alleviates cardiac hypertrophy in pressure overload mice. Supplementary Figure 2: FTZ reduces cardiac fibrosis in pressure overload mice. Supplementary Figure 3: CCK-8 kits for cell viability detection. Supplementary Figure 4: effects of FTZ on cell apoptosis. Supplementary Figure 5: FTZ reduces matrix metalloproteinases *in vitro*. Supplementary Figure 6: TGF*β*1 agonist reverses FTZ inhibition of cardiac fibroblast activation. Supplementary materials associated with this article can be found in the online version.
