# Supplementary material for: The Traditional Chinese Medicine Formula FTZ Protects against Cardiac Fibrosis by Suppressing the TGFβ1-Smad2/3 Pathway
Source: Evid Based Complement Alternat Med. 2022 Apr 19;2022:5642307. doi: 10.1155/2022/5642307 (PMC9042631; doi:10.1155/2022/5642307)
Supplement: Supplementary Materials — Supplementary Figure 1: FTZ alleviates cardiac hypertrophy in pressure overload mice. Supplementary Figure 2: FTZ reduces cardiac fibrosis in pressure overload mice. Supplementary Figure 3: CCK-8 kits for cell viability detection. Supplementary Figure 4: effects of FTZ on cell apoptosis. Supplementary Figure 5: FTZ reduces matrix metalloproteinases in vitro. Supplementary Figure 6: TGFβ1 agonist reverses FTZ inhibition of cardiac fibroblast activation. Supplementary materials associated with this article can be found in the online version. [file 5642307.f1.docx]

**The Traditional Chinese Medicine Formula FTZ Protects Against Cardiac Fibrosis by Suppressing the TGFβ1-Smad2/3 pathway**

**Yue Zhang^a,1^, Dongwei Wang^a,1^, Kaili Wu^a^, Xiaoqi Shao^a^, Hongtao Diao^a^, Zhiying Wang^a^, Mengxian Sun^a^, Xueying Huang^a^, Yun Li^a^, Xinyuan Tang^a^, Meiling Yan^a^ and Jiao Guo^b,c,d,e,*^**

^a^ Center for Drug Research and Development, Guangdong Pharmaceutical University, Guangzhou, 510006, China.

^b^ Guangdong Metabolic Diseases Research Center of Integrated Chinese and Western Medicine; Guangzhou, 510006, China.

^c^ Key Laboratory of Glucolipid Metabolic Disorder, Ministry of Education of China.

^d^ Institute of Chinese Medicine, Guangdong Pharmaceutical University; Guangzhou, 510006, China.

^e^ Guangdong TCM Key Laboratory for Metabolic Diseases, Guangzhou 510006, China.

^*^ Corresponding author.

Jiao Guo, 280 Wai Huan Dong Road, Guangdong Pharmaceutical University, Guangzhou Higher Education Mega Center, Guangzhou 510006, China. Phone: +86 20 39352818; E-mail: gyguoyz@163.com

^1^ These authors contributed equally to this work.

**Supplementary materials**

**Methods and Materials**

**S1.1. Chemical treatments**

TGFβ1 agonist was purchased from Peprotech (0521209), and TGFβ1inhibitor (ITD-1) was purchased form AdooQ Bioscience (A12924). The cells were treated with 10 ng/ml TGFβ1 agonist or 5μM ITD-1 for 24 h, then were used for further analysis.

**S1.2. TUNEL staining**

After drug treatment, CFs were fixed in 4% paraformaldehyde and treated with 0.3% Triton X-100 for 20 min, then stained according to the manufacturers’ instructions (Roche 52733700). Cell apoptosis was observed and images were photographed using a fluorescence microscope (Olympus Optics, Tokyo, Japan). The Tunel positive cell was calculated by using Image J software.

**S1.3. Hydroxyproline (HYP) content**

The content of hydroxyproline in the heart of mice was determined by the ELISA kits (ELK Biotechnology, ELK9115). A total of 20 mg of left ventricular cardiac tissues was obtained from each group, and then was grinded with PBS. The hydroxyproline content was measured according to the manufacturers’ instructions. The absorbance of final solution was determined at 450 nm.

**Result**

**Figure S1**

**
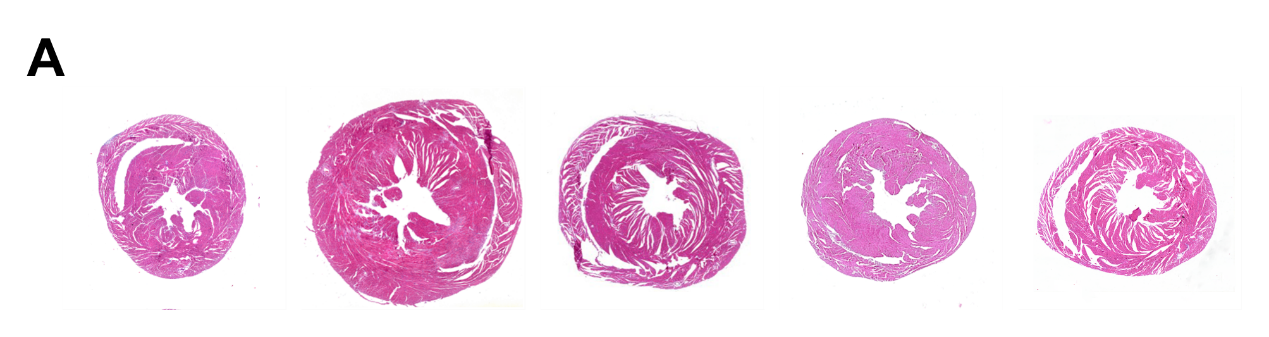
**

**Figure S1. FTZ alleviates cardiac hypertrophy in pressure overload mice.** (A) H&E staining results of cardiac tissue represented. Scale bars, 2 mm. n = 3 per group.

**Figure S2**


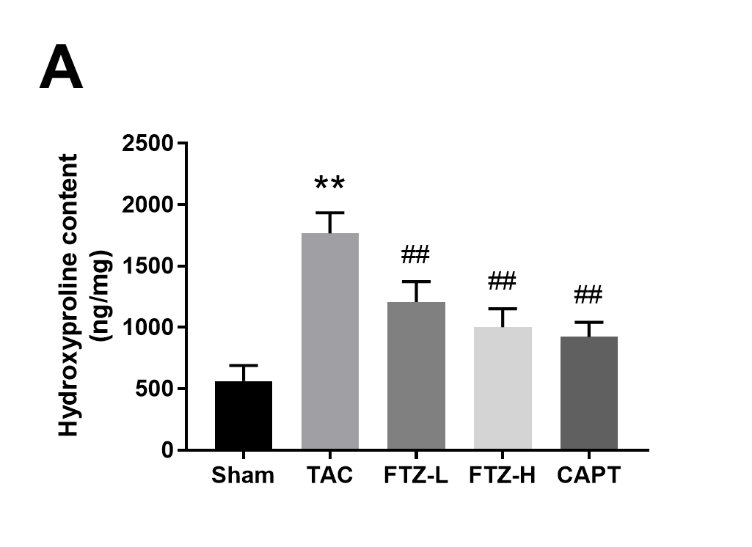


**Figure S2. FTZ reduces cardiac fibrosis in pressure overload mice.** (A) The content of hydroxyproline was analyzed by ELISA. n = 6 per group. *^**^P*<0.01 vs the Sham group; *^##^P*<0.01 vs the TAC group.

**Figure S3**


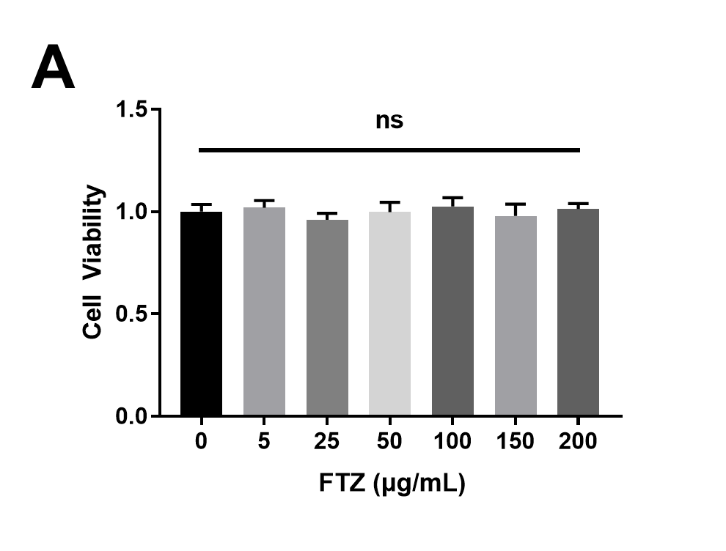


**Figure S3. CCK-8 kits for cell viability detection.** (A) Effects of different concentrates of FTZ on viability of cardiac fibroblasts. n =8 per group.

**Figure S4**


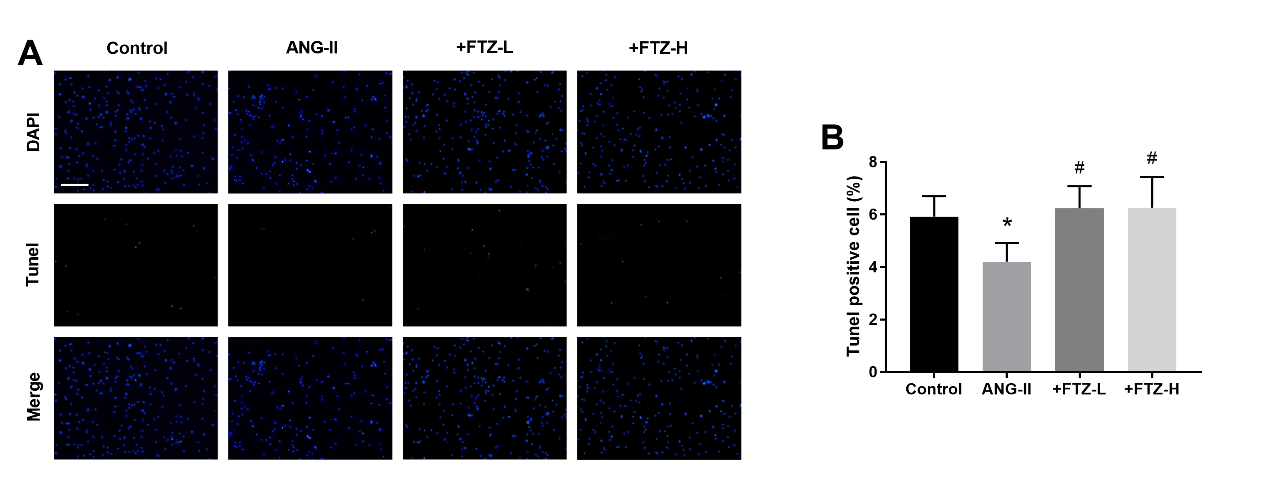


**Figure S4. Effects of FTZ on cell apoptosis.** (A-B) Representative images of Tunel staining (green) and DAPI (blue). Scale bar indicates 100 μm. n =5 per group.

**Figure S5**


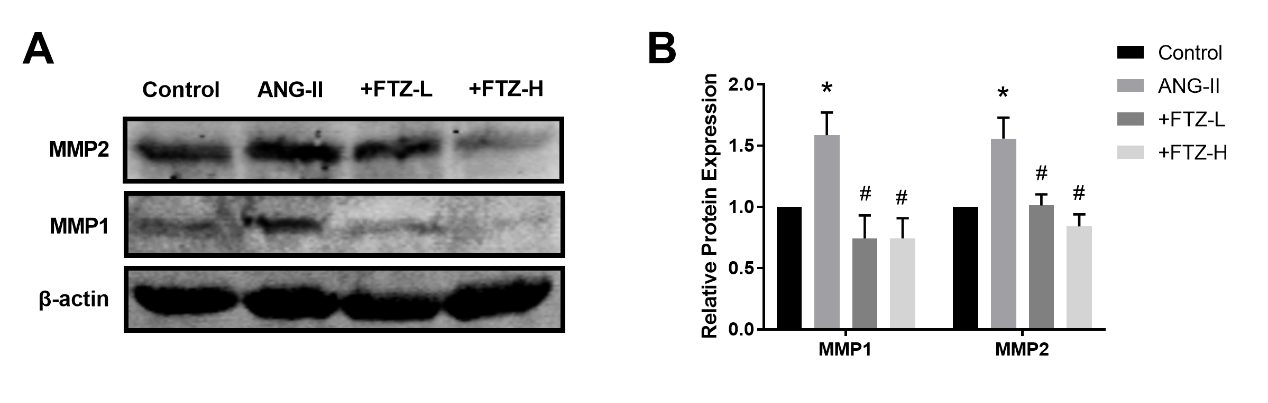


**Figure S5. FTZ reduces matrix metalloproteinases in vitro.** (A-B) The expression of MMP1 and MMP2 was analyzed by Western blot. n =3 per group. Data represent the mean ±SEM. *^*^P*<0.05 vs the Control group; *^#^P*<0.05 vs the Ang-II group.

**Figure S6**


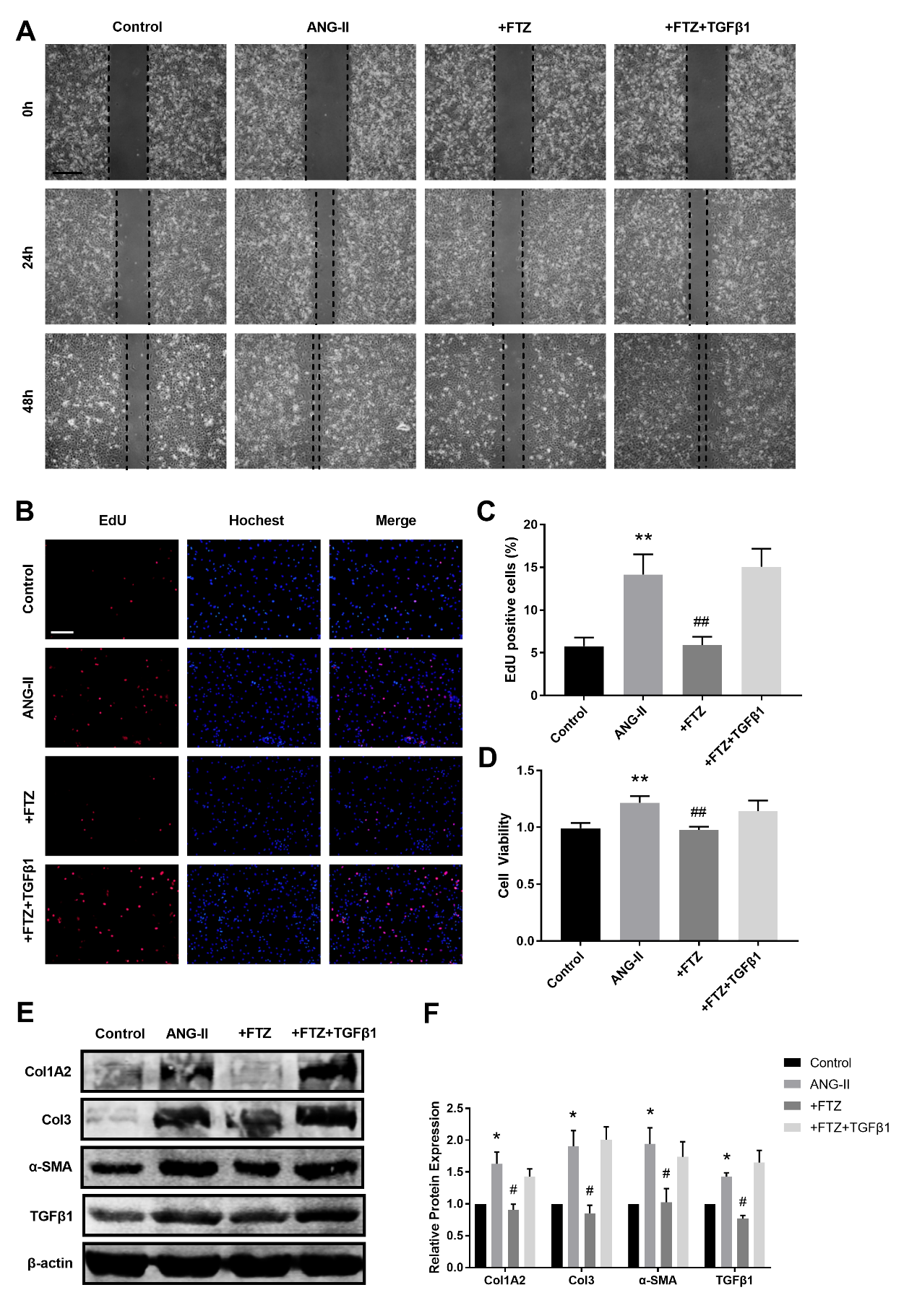


**Figure S6. TGFβ1 agonist reverses FTZ inhibition of cardiac fibroblast activation.** (A) Representative images of wound heaing assay. Scale bars, 200 μm. n =5 per group. (B-C) Representative images of EdU staining showing proliferation cells (stained in red). Nuclei that double-labeled with EdU (red) and Hoechst 33342 (blue) were considered to be new proliferative cells. Scale bar indicates 100 μm. n =5 per group. (D) Cell viability were measured with a CCK-8 assay. n =8 per group. (E-F) The expression of Col1A2, Col3, α-SMA and TGFβ1 was analyzed by Western blot. n =3 per group. Data represent the mean ±SEM. *^*^P*<0.05, *^**^P*<0.01 vs the Control group; *^#^P*<0.05, *^##^P*<0.01 vs the Ang-II group.
